# QuEChERS-液相色谱-串联质谱法测定中草药中22种三唑类农药残留

**DOI:** 10.3724/SP.J.1123.2022.08005

**Published:** 2023-04-08

**Authors:** Jiao WANG, Tong WU, Xinquan WANG, Zhenzhen LIU, Hao XU, Zhiwei WANG, Shanshan DI, Huiyu ZHAO, Peipei QI

**Affiliations:** 省部共建农产品质量安全危害因子与风险防控国家重点实验室, 浙江省农业科学院农产品质量安全与营养研究所, 浙江 杭州 310021; State Key Laboratory for Managing Biotic and Chemical Threats to the Quality and Safety of Agro-products, Institute of Agro-product Safety and Nutrition, Zhejiang Academy of Agricultural Sciences, Hangzhou 310021, China

**Keywords:** 液相色谱-串联质谱法, 三唑类农药残留, 浙八味, 中草药, 羧基化多壁碳纳米管, liquid chromatography-tandem mass spectrometry (LC-MS/MS), triazole pesticide residues, Zhebawei, Chinese herbal medicines, carboxylated multi-wall carbon nanotubes

## Abstract

建立了“浙八味”中草药中22种三唑类杀菌剂农药残留的简便、快速和精准分析方法。以白术为代表性基质,对QuEChERS前处理方法进行改良,样品经乙腈萃取后,选取10 mg羧基化多壁碳纳米管和20 mg C18组合作为净化吸附剂,并采用液相色谱-串联质谱进行分析;对建立的方法进行了系统考察,糠菌唑、氟环唑和乙环唑的线性范围为2.5~200 μg/L,其余农药的线性范围为1~200 μg/L,相关系数(*R*)均大于0.99;在10、20、100和200 μg/kg的添加水平下平均回收率为77.0~115%,相对标准偏差(RSD)均低于9.4%;本方法的检出限为1~2.5 μg/kg,定量限为10~20 μg/kg;通过添加回收试验验证该方法对“浙八味”其他中草药的适用性,目标农药在不同基质中的平均回收率为76.4%~123%, RSD均低于12.2%。利用该方法检测了市售30份“浙八味”中草药中三唑类农药残留,结果显示,浙贝母和杭白菊中有三唑类农药检出,其中浙贝母中均检出苯醚甲环唑,含量为41.4~110 μg/kg,杭白菊中检出苯醚甲环唑、腈菌唑、三唑醇和丙环唑,含量为16.1~250 μg/kg。所建立的方法操作简便快速,方法灵敏度高,重复性好,可满足“浙八味”中三唑类杀菌剂准确定量分析的要求。

## Abstract (en)

Eight well-known herbals in Zhejiang Province, Zhebawei, are commonly used as traditional Chinese herbal medicines owing to their rich active ingredients. However, the unavoidable use of pesticides during agricultural production has led to pesticide residue problems in these herbs. In this study, a simple, rapid, and accurate method was established to determine 22 triazole pesticide residues in Zhebawei. An improved QuEChERS method was used for sample pretreatment, and *Rhizoma Atractylodis Macrocephalae* was used as a representative sample. The sample was extracted with acetonitrile to eliminate some polar and nonpolar compounds, pigments, and other impurities, and the purification effects of multiwalled carbon nanotubes (MWCNTs), amino-modified multiwalled carbon nanotubes (MWCNTs-NH_2_), carboxylated multiwalled carbon nanotubes (MWCNTs-COOH), crosslinked polyvinylpyrrolidone (PVPP), zirconium dioxide (ZrO_2_), 3-(*N*,*N*-diethylamino)-propyltrimethoxysilane (PSA), octadecyl (C18), and graphitized carbon black (GCB) were compared. MWCNTs-COOH and C18 were selected as the purification adsorbents, and their dosages were systematically optimized. The combination of 10 mg of MWCNTs-COOH and 20 mg of C18 was eventually selected as the purification adsorbents. Liquid chromatography-tandem mass spectrometry (LC-MS/MS) was used for analysis, and box graphs were plotted to present the dispersion of each group of recoveries, thus enabling the identification of the data outliers, dispersion distribution, and data symmetry. The established method was systematically verified and showed good linearity over the concentration range of 1-200 μg/L (except for bromuconazole, epoxiconazole, and etaconazole) with correlation coefficients >0.99. The average recoveries of the 22 pesticides at spiked levels of 10, 20, 100, and 200 μg/kg were in the range of 77.0%-115% with relative standard deviations (RSDs) <9.4%. The limits of detection and quantification were 1-2.5 μg/kg and 10-20 μg/kg, respectively. The applicability of the developed method to other herbals was investigated at 100 μg/kg, and the average recoveries of the target pesticides in different matrices ranged from 76.4% to 123% with RSDs <12.2%. Finally, the method established was used to detect triazole pesticide residues in 30 actual Zhebawei samples. The results showed that triazole pesticides were present in *Bulbus Fritillariae Thunbergii* and *Dendranthema Morifolium*. Difenoconazole was detected in *Bulbus Fritillariae Thunbergii* at contents ranging from 41.4 μg/kg to 110 μg/kg, while difenoconazole, myclobutanil, triadimenol and propiconazole were detected in *Dendranthema Morifolium* at contents ranging from 16.1 μg/kg to 250 μg/kg. The established method can meet the requirements for the accurate quantitative analysis of triazole fungicides in Zhebawei.

浙江省特色药材“浙八味”,白术(*Rhizoma Atractylodis Macrocephalae*)、杭白菊(*Dendranthema Morifolium*)、延胡索(*Rhizoma Corydalis*)、郁金(*Radix Curcumae*)、玄参(*Radix Scrophulariae*)、浙贝母(*Bulbus Fritillariae Thunbergii*)、麦冬(*Radix Ophiopogonis*)和白芍(*Radix Paeoniae Alba*),为大宗常用类中草药。“浙八味”含有丰富的活性成分如多糖、酚类、生物碱、黄酮和挥发油等化合物^[1⇓-3]^,具有增强免疫力、抗氧化、消炎等作用,市场需求不断增大^[4⇓-6]^。在“浙八味”种植过程中,为了保证产量,杀菌剂的使用不可避免。杀菌剂按照原料来源可分为有机磷杀菌剂、有机硫杀菌剂、酰胺类杀菌剂和三唑类杀菌剂等,其中三唑类杀菌剂应用广泛,但其化学性质稳定,不易降解^[7]^,易造成农药残留。 目前尚无完善的检测技术可用来分析“浙八味”中的三唑类杀菌剂残留^[8,9]^,因此,为了严控“浙八味”药材的质量安全问题,急需开发快速、简便、精准的三唑类农药残留分析方法。

QuEChERS方法具有操作简便、耗时短、有机溶剂消耗少、成本低等优势,已广泛应用于多农药残留分析^[10]^。其中,基于分散固相萃取技术的净化过程是决定方法准确度和灵敏度的关键。净化过程利用净化吸附剂对杂质成分进行吸附、分离,将目标分析物保留在提取液中,从而做到去除杂质,纯化目标分析物的目的。目前,*N*-丙基乙二胺(PSA)、十八烷基硅烷(C18)和石墨化炭黑(GCB )是常用的净化吸附剂,它们分别对一些极性化合物、非极性化合物和色素有很好的去除作用^[11]^。然而,对于成分复杂的基质,传统的吸附剂不能达到理想的净化效果,杂质成分在提取过程中可能与目标分析物一起被提取,影响目标分析物定量结果的准确性,且易对检测仪器造成污染,从而缩短仪器的使用寿命,增加仪器的维护成本^[12]^。近几年,针对复杂基质中杂质成分的净化,研究人员相继研发出多种新型净化吸附剂用于样品净化。例如,二氧化锆(ZrO_2_)对牛油果、食用油等一些高脂样品具有很好的净化效果^[13,14]^;聚乙烯聚吡咯烷酮(PVPP)对茶叶中的多酚类物质有很好的吸附性能^[15]^。因此,根据基质特点选择净化吸附剂是实现复杂样品中农药残留精准分析的关键。多壁碳纳米管(MWCNTs)具有比表面积较大、结构独特且易于功能化的优势^[16]^, MWCNTs以及其功能化产品^[17]^对黄酮、酚类和色素等极性化合物展现出良好的吸附能力,已被广泛用于水果、蔬菜和茶叶等复杂基质中的农药残留分析^[18⇓-20]^; C18作为传统净化吸附剂可吸附非极性油类化合物成分,与新型净化吸附剂共同组合常应用于样品前处理中^[21]^。因此,针对含有多种极性和非极性化合物的“浙八味”复杂基质,本研究尝试使用羧基化MWCNTs(MWCNTs-COOH)和C18共同作为净化吸附剂,探索其对“浙八味”中杂质的净化效果。

本研究以22种三唑类杀菌剂为研究对象,以白术为代表性基质,样品经乙腈萃取后,利用MWCNTs-COOH和C18共同作为净化吸附剂,通过探究其用量对净化效果的影响,结合液相色谱-串联质谱法(LC-MS/MS),建立了适用于8种中草药基质中三唑类杀菌剂农药残留检测的方法。

## 1 实验部分

### 1.1 仪器、试剂与材料

LC-30AD液相色谱仪和8050三重四极杆质谱仪(日本Shimadzu公司); Biofuge Primo R型台式离心机(美国Thermo公司); BS224S电子天平和BSA2202S电子天平(感量分别为0.0001 g和0.01 g)(德国Sartorius公司); VX-Ⅲ多管涡旋振荡器(北京踏锦科技有限公司); Milli-Q超纯水器(美国Millipore公司)。

色谱纯甲醇、乙腈(德国Merck公司);色谱纯甲酸铵(美国天地有限公司); PSA、GCB和C18(天津博纳艾杰尔科技有限公司); MWCNTs、氨基化多壁碳纳米管(MWCNTs-NH_2_)和MWCNTs-COOH(南京先丰纳米材料科技有限公司); PVPP(广东康达生物科技有限公司); ZrO_2_(上海阿拉丁生化科技股份有限公司);无水硫酸镁(MgSO_4_)和氯化钠(NaCl)(上海凌峰化学试剂有限公司)。

农药标准品及标准溶液:粉唑醇(98.2%,纯度,下同)、三唑酮(99.5%)、环丙唑醇(97.5%)、三唑醇(98.7%)、戊唑醇(1000 mg/L,溶剂为丙酮)、己唑醇(99.3%)购自上海农药研究所;氧环唑(99.5%)、腈菌唑(99.6%)、糠菌唑(99.8%)、四氟醚唑(100 mg/L,溶剂为正己烷)、硅氟唑(98.7%)、氟环唑(98.5%)、腈苯唑(99.0%)、乙环唑(99.9%)、氟硅唑(99.5%)、烯效唑(99.8%)、戊菌唑(98.1%)、丙环唑(99.0%)、联苯三唑醇(99.0%)、叶菌唑(98.5%)、苯醚甲环唑(98.8%)、种菌唑(98.0%)购自农业部环境保护科研监测所。

### 1.2 标准溶液的配制

标准储备溶液:准确称取固体农药标准品各10 mg(精确至0.0001 g)至10 mL容量瓶中,用甲醇溶解并定容,配制成质量浓度为1000 mg/L的标准储备液,在-4 ℃下保存。

混合标准工作溶液:分别吸取一定量农药标准溶液及储备液至25 mL容量瓶中,用甲醇定容,得到5 mg/L的混合标准工作溶液,在-4 ℃下贮存。

### 1.3 实验条件

#### 1.3.1 样品前处理

杭白菊样品采自浙江省桐乡市;其余7种中药材购于百姓中药材和中药材市场。将样品高速研磨成粉,置于干燥器中避光保存。准确称取2 g样品于50 mL离心管中,加入8 mL超纯水(麦冬和菊花加入12 mL超纯水)涡旋振荡1 min使样品充分浸湿,加入5 mL乙腈涡旋振动1 min,再加入6 g MgSO_4_和1.5 g NaCl,涡旋振荡1 min后以7000 r/min离心3 min。

转移1 mL上清液至预先装有10 mg MWCNTs-COOH、20 mg C18和150 mg MgSO_4_的2 mL离心管中,涡旋振荡1 min,以7000 r/min离心3 min,取上清液过0.22 μm有机滤膜到进样小瓶中,待测。

#### 1.3.2 色谱条件

色谱柱为ACE Excel C18(100 mm×2.1 mm, 1.7 μm);柱温箱温度为35 ℃;流动相:A为5 mmol/L甲酸铵水溶液,B为甲醇,总流速为0.3 mL/min;采用总运行时间为10 min的梯度洗脱程序:0~1.00 min, 5%B~40%B; 1.00~3.00 min, 40%B~80%B; 3.00~5.00 min, 80%B~95%B; 5.00~8.00 min, 95%B; 8.01 min, 5%B,保持1.99 min;进样体积为2 μL。

#### 1.3.3 质谱条件

采用电喷雾离子源,正离子模式,离子源电压为4000 V,离子源温度为300 ℃;碰撞气为氩气;雾化气和干燥气均为氮气,流速分别是3 L/min和10 L/min;加热气为空气,流量设置为10 L/min;离子采集模式为多反应监测,目标农药的详细离子对信息及质谱参数见表1。

**表1:** 22种三唑类杀菌剂的质谱分析参数

Compound	Retention time/min	Parent ion (m/z)	Product ions (m/z)	Q^1^ pre bias/V	Collision energy/eV	Q^3^ pre bias/V
Azaconazole (氧环唑)	4.143	300.00	159.00^*^, 231.00	-15, -15	-27, -17	-29, -23
Bitertanol (联苯三唑醇)	4.995	338.20	269.15^*^, 99.10	-17, -17	-9, -15	-29, -18
Bromuconazole (糠菌唑)	4.491	377.90	158.90^*^, 70.00	-19, -19	-28, -23	-30, -30
Cyproconazole (环丙唑醇)	4.464	292.10	70.05^*^, 125.05	-30, -30	-20, -30	-27, -22
Difenoconazole (苯醚甲环唑)	5.167	406.10	251.00^*^, 337.05	-30, -30	-25, -17	-27, -24
Epoxiconazole (氟环唑)	4.567	330.10	121.20^*^, 141.10	-17, -17	-21, -18	-22, -25
Etaconazole (乙环唑)	4.615	327.90	159.20^*^, 123.15	-16, -16	-28, -55	-18, -14
Fenbuconazole (腈苯唑)	4.601	336.90	125.05^*^, 70.00	-26, -26	-27, -20	-25, -28
Flusilazole (腈苯唑)	4.647	316.10	165.10^*^, 247.10	-30, -30	-29, -18	-30, -27
Flutriafol (粉唑醇)	3.992	302.10	123.00^*^, 109.00	-15, -15	-28, -31	-22, -19
Hexaconazole (己唑醇)	5.031	314.10	70.20^*^, 159.15	-15, -15	-21, -29	-28, -30
Ipconazole (种菌唑)	5.321	334.00	70.30^*^, 125.20	-23, -23	-23, -47	-14, -14
Metconazole (叶菌唑)	5.015	320.00	70.10^*^, 125.05	-23, -23	-40, -37	-28, -23
Myclobutanil (腈菌唑)	4.415	289.10	70.05^*^, 125.05	-30, -30	-21, -30	-28, -22
Penconazole (戊菌唑)	4.876	284.10	70.00^*^, 159.00	-14, -14	-17, -27	-27, -30
Propiconazole (丙环唑)	4.942	342.05	159.10^*^, 205.10	-17, -17	-30, -18	-29, -21
Simeconazole (硅氟唑)	4.534	294.10	70.05^*^, 135.05	-15, -15	-21, -21	-28, -24
Tebuconazole (戊唑醇)	4.835	308.10	70.10^*^, 125.00	-22, -22	-22, -38	-27, -23
Tetraconazole (四氟醚唑)	4.495	372.00	159.05^*^, 70.20	-27, -27	-31, -24	-29, -27
Triadimefon (三唑酮)	4.408	294.10	69.15^*^, 197.05	-21, -21	-22, -15	-26, -21
Triadimenol (三唑醇)	4.487	296.10	70.05^*^, 99.15	-15, -15	-11, -15	-29, -17
Uniconazole (烯效唑)	4.689	292.10	70.10^*^, 125.00	-21, -21	-24, -28	-27, -23

*Quantitative ion.

## 2 结果与讨论

### 2.1 分析方法的建立

本方法中净化剂的选择与用量是决定样品净化效果和分析方法准确度的关键因素,因此,方法优化中,通过考察不同因素对三唑类农药回收率的影响来筛选最佳的实验条件,以白术样品为代表性基质进行所有的方法优化实验,各农药的添加水平均为50 μg/kg。根据测定的实际浓度与基质匹配标准溶液浓度的百分比值计算回收率,回收率的最佳效果是趋近100%,在农药残留分析中可接受的回收率范围是70%~120%。净化吸附剂筛选过程中,样品经乙腈萃取离心后,准确移取1 mL上清液转移至预装有不同净化剂(MWCNTs、MWCNTs-NH_2_、MWCNTs-COOH、PVPP、ZrO_2_、PSA、C18或GCB)和150 mg硫酸镁的2 mL离心管中,样品经净化离心后,过膜采用LC-MS/MS分析;净化吸附剂用量优化过程中,样品经乙腈萃取离心后,将1 mL的白术样品提取溶液加到预先装有150 mg MgSO_4_和不同剂量MWCNTs-COOH(10、20、30、40和50 mg)或C18(10、20、30、40和50 mg)的2 mL离心管中,经净化后分析,计算回收率。本研究利用箱形图显示每组回收率数据的分散情况,能够直观地显示数据的异常值、分布的离散程度以及数据的对称性。箱形图共由7个数值点构成,分别是最小值(下边缘)、 25%分位数(箱型底部)、中位数(箱内横线)、平均值(箱内小方框)、 75%分位数(箱型上部)、最大值(上边缘)和异常值(箱外小方框)。

#### 2.1.1 净化吸附剂筛选

比较了MWCNTs、MWCNTs-NH_2_、MWCNTs-COOH、PVPP、ZrO_2_、PSA、C18和GCB净化吸附剂各30 mg对目标农药回收率的影响。如图1所示,在MWCNTs、MWCNTs-NH_2_、MWCNTs-COOH、PVPP、ZrO_2_、PSA、C18和GCB为净化吸附剂时所有农药的平均回收率分别为108%、104%、95.2%、132%、117%、119%、108%和116%。MWCNTs-NH_2_、MWCNTs-COOH和C18的平均回收率更接近100%,且使用MWCNTs-COOH时农药回收率均在70%~120%范围内,但使用MWCNTs-NH_2_和C18时分别有3个(糠菌唑、三唑酮和四氟醚唑)和1个(腈菌唑)农药回收率高于120%。因此本实验拟选择MWCNTs-COOH和C18为后续的净化吸附剂。

**图1:**
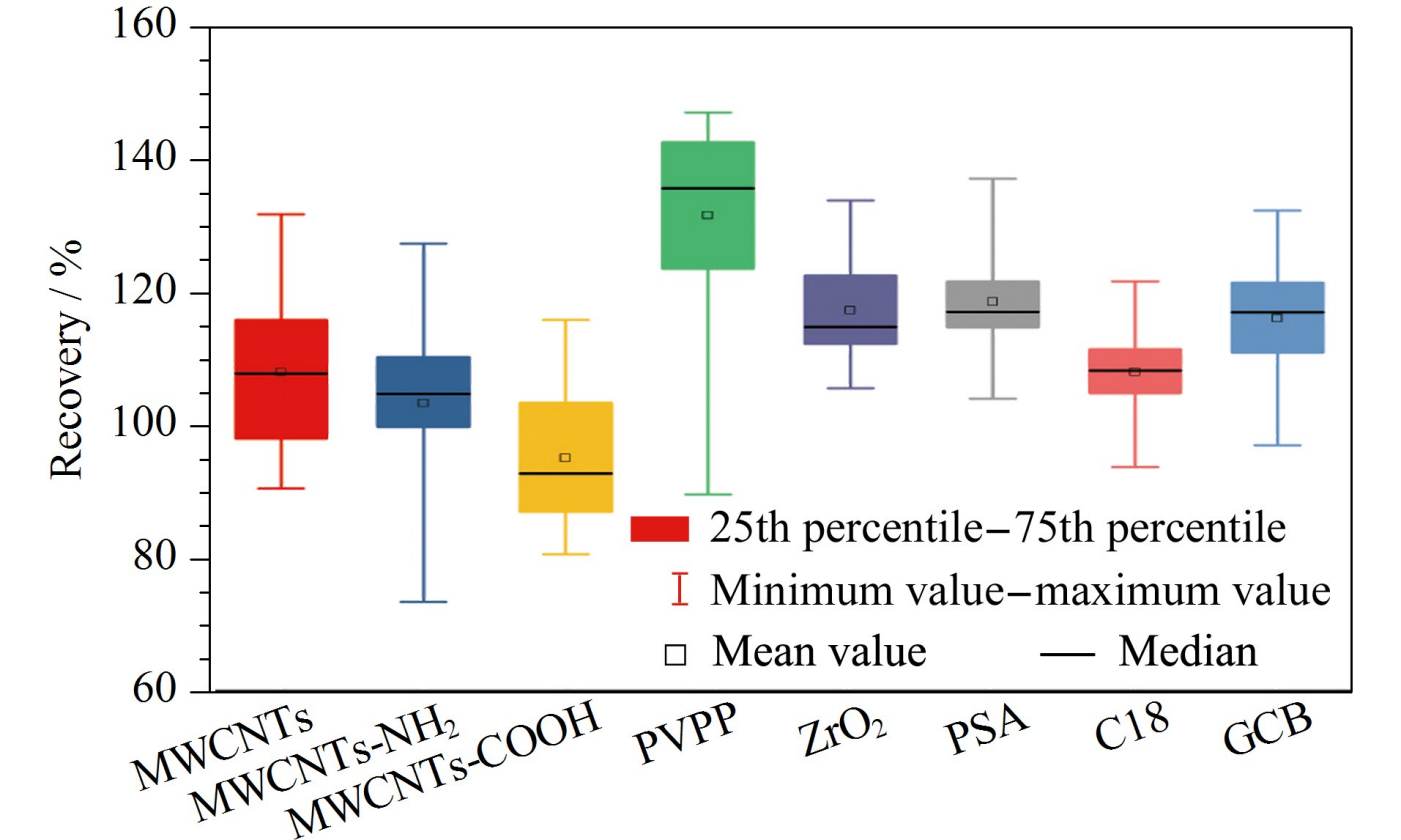
不同净化吸附剂对22种农药回收率的影响

#### 2.1.2 吸附剂用量优化

本研究进一步考察了吸附剂MWCNTs-COOH的用量对目标农药回收率的影响。如图2a所示,使用不同量MWCNTs-COOH吸附剂时,平均回收率分别为105%、107%、101%、100%和87.4%,相差不大,但与其他条件相比,使用10 mg MWCNTs-COOH时所有农药的回收率更加集中,此外发现部分农药(环丙唑醇、腈苯唑、戊唑醇、烯效唑、叶菌唑和种菌唑)的回收率随着MWCNTs-COOH用量增加而降低(图2b),可能由于过多的MWCNTs-COOH会对这些农药有一定吸附作用,因此将MWCNTs-COOH的用量定为10 mg。

**图2:**
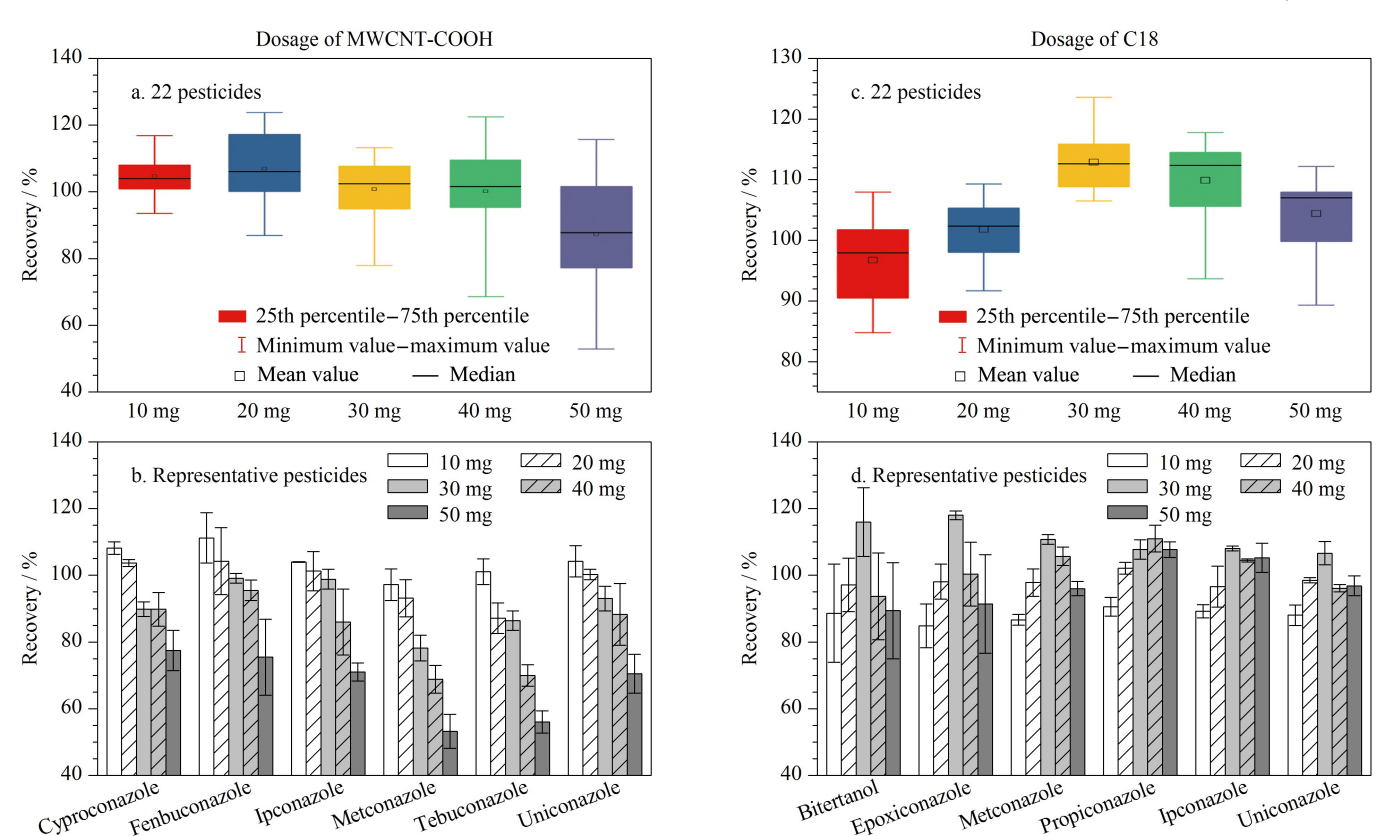
MWCNTs-COOH用量对(a)22种农药回收率和(b)代表性农药回收率的影响,以及C18用量对(c)22种农药回收率和(d)代表性农药回收率的影响

在使用10 mg MWCNTs-COOH的基础上,考察了添加不同量的C18对农药回收率的影响。使用不同量的C18吸附剂时,22种农药的平均回收率分别为96.8%、102%、113%、110%和104%,可见随着C18用量的增加,所有农药的平均回收率呈先上升后降低的趋势(图2c);在使用20 mg C18时,所有农药的回收率更加集中且接近100%。此外,受C18用量影响较大的农药(联苯三唑醇、氟环唑、叶菌唑、丙环唑、种菌唑和烯效唑)的回收率在20 mg C18时更接近100%(图2d),因此将C18的用量定为20 mg。

为验证MWCNTs-COOH和C18对样品的净化效果,对比了未经净化和经吸附剂净化后的白术样品基质溶液的色泽变化及目标物的谱图变化。由图3可以看出,在使用MWCNTs-COOH和C18吸附剂后,白术基质的色素成分逐次减少,样品得以净化;同时,对比代表性农药联苯三唑醇、腈苯唑和戊菌唑的提取离子流色谱图(见图4)可以看出,未经净化时色谱图中杂质峰较多,经净化后杂质峰相对减弱,证明白术样品经净化吸附剂净化后可显著降低基体杂质对农药的定性定量分析准确度的影响。

**图3:**
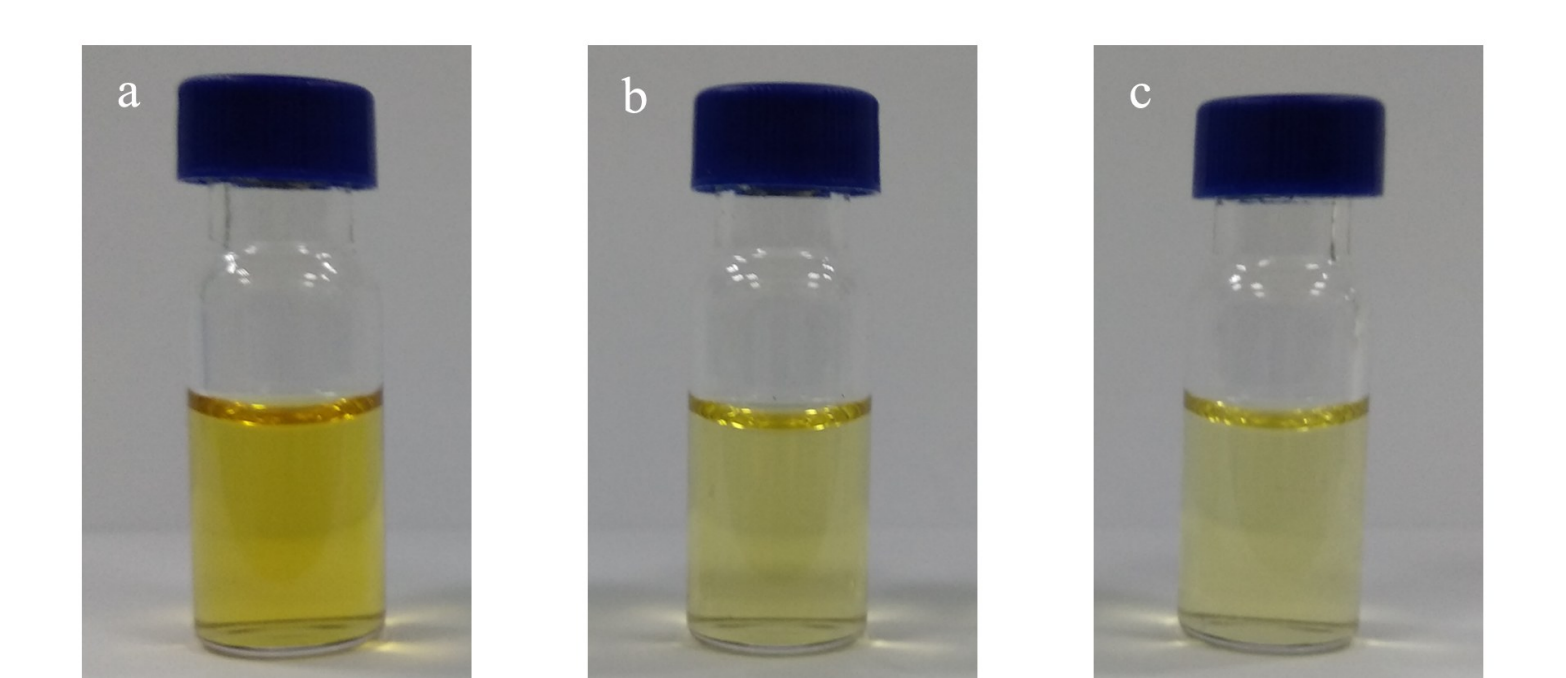
白术样品基质的颜色变化

**图4:**
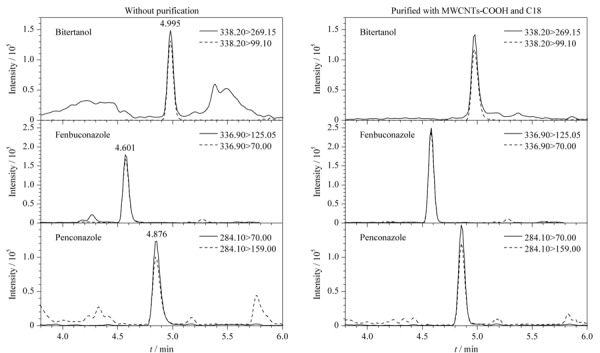
白术未经净化和经10 mg MWCNTs-COOH+20 mg C18净化时联苯三唑醇、腈苯唑和戊菌唑的提取离子流色谱图

### 2.2 方法学考察

取适量混合标准工作溶液用乙腈或经提取净化后的白术空白样品基质溶液稀释成一定浓度的标准溶液,现用现配。配制质量浓度为1、2.5、5、10、25、50、100、150、200 μg/L的系列标准溶液,以农药质量浓度(*x*)为横坐标,色谱峰面积(*y*)为纵坐标,绘制校正曲线。采用基质匹配校正曲线和溶剂校正曲线的斜率比值来评估基质效应,斜率比等于1无基质效应;斜率比小于1呈基质抑制效应;斜率比大于1呈基质增强效应^[22]^。线性关系见附表1(www.chrom-China.com),基质效应见表2。结果表明,糠菌唑、氟环唑和乙环唑的线性范围为2.5~200 μg/L,其余农药的线性范围为1~200 μg/L,相关系数(*R*)均大于0.99,表明各农药在相应的范围内均呈现良好的线性关系;22种农药的基质效应为0.12~0.64,均为基质抑制效应,表明虽然所选净化吸附剂对目标农药的回收率和色素方面有改善作用,但中草药基质复杂,对目标农药的定量分析仍有一定影响,因此需要使用基质匹配标准溶液进行定量分析。

**表2:** 白术样品中22种三唑类杀菌剂的基质效应、4个水平下的加标回收率、定量限和检出限

Analyte	Matrix effect	Recoveries (RSDs)/%								LOQ/ (μg/kg)	LOD/ (μg/kg)
		10 μg/kg		20 μg/kg		100 μg/kg		200 μg/kg			
Azaconazole	0.2	115	(3.8)	103	(4.1)	105	(2.0)	101	(5.6)	10	2.0
Bitertanol	0.6	98.3	(3.3)	108	(4.4)	109	(2.4)	109	(3.5)	10	2.0
Bromuconazole	0.3	-		104	(9.2)	109	(2.2)	96.9	(4.1)	20	2.5
Cyproconazole	0.2	109	(4.2)	108	(2.5)	106	(1.5)	105	(5.4)	10	2.0
Difenoconazole	0.3	102	(4.0)	101	(4.7)	109	(3.0)	107	(3.2)	10	2.0
Epoxiconazole	0.2	-		99.0	(2.8)	105	(1.7)	103	(9.4)	20	2.5
Etaconazole	0.2	-		111	(3.4)	103	(2.1)	108	(4.8)	20	2.5
Fenbuconazole	0.3	109	(5.8)	107	(6.6)	107	(3.5)	103	(5.4)	10	1.0
Flusilazole	0.2	111	(7.4)	112	(4.6)	107	(1.6)	106	(3.6)	10	2.0
Flutriafol	0.4	115	(4.1)	110	(3.4)	108	(2.4)	109	(4.1)	10	2.0
Hexaconazole	0.4	110	(2.4)	103	(6.8)	108	(2.6)	111	(3.3)	10	1.5
Ipconazole	0.2	92.6	(8.9)	93.9	(0.9)	108	(3.4)	104	(7.2)	10	2.0
Metconazole	0.3	97.7	(2.2)	93.0	(6.8)	103	(1.9)	92.7	(2.4)	10	2.0
Myclobutanil	0.4	104	(2.9)	101	(2.6)	99.1	(1.5)	104	(5.1)	10	1.0
Penconazole	0.1	107	(3.8)	98.9	(8.1)	110	(2.2)	107	(9.2)	10	1.0
Propiconazole	0.3	102	(2.7)	94.0	(5.8)	111	(2.0)	106	(1.6)	10	2.0
Simeconazole	0.1	111	(4.0)	110	(2.2)	109	(4.4)	101	(8.0)	10	2.0
Tebuconazole	0.2	101	(3.4)	98.7	(2.6)	104	(4.0)	104	(3.7)	10	2.0
Tetraconazole	0.3	114	(8.2)	112	(7.6)	104	(1.7)	107	(5.9)	10	2.0
Triadimefon	0.3	77.0	(5.0)	98.0	(4.7)	102	(1.9)	103	(7.2)	10	1.5
Triadimenol	0.5	108	(6.6)	113	(4.5)	111	(1.6)	107	(6.0)	10	1.0
Uniconazole	0.2	95.9	(6.1)	99.0	(4.2)	98.7	(1.1)	98.0	(2.1)	10	2.0

-: not detected.

利用加标回收试验对方法的准确度和精密度进行验证,同时确定方法的定量限(LOQ)。向空白白术样品中分别添加目标农药,使其含量水平分别为10、20、100和200 μg/kg,每个含量水平做6次重复,计算平均回收率以评估方法的准确度,以6次重复的相对标准偏差(RSD)评估方法的精密度。添加回收试验结果见表2。在10 μg/kg添加水平下,除糠菌唑、氟环唑和乙环唑未被检出外,其他农药的回收率为77.0%~115%,并且在20、100和200 μg/kg添加水平下各农药的回收率均在92.7%~113%范围内,4个添加水平下农药的RSD均小于9.4%。参考欧盟的农药残留分析方法验证文件SANTE/12682/2019, LOQ确定为在添加回收试验中能够满足回收率和精密度要求的最低添加水平,因此糠菌唑、氟环唑和乙环唑的LOQ为20 μg/kg,其他农药的LOQ为10 μg/kg。

方法的LOD可通过在空白样品基质溶液中添加已知浓度分析物,然后根据能够可靠检测出分析物的最低浓度值来确定。在样品空白中加入一系列不同浓度的分析物,每个浓度点需要进行7次测试^[23]^。据此,制备0.2、0.4、0.6、0.8、1和2.5 μg/L系列质量浓度的基质匹配标准溶液进行检测(重复7次),22种三唑类农药的最低检出质量浓度为0.4~1 μg/L,考虑到本方法样品制备过程稀释倍数为2.5,因此样品中农药的LOD为1.0~2.5 μg/kg,见表2。目前三唑类杀菌剂在药用植物上的最大残留限量标准还不完善,因此参考植物源性食品的残留限量标准(GB 2763-2021)。除糠菌唑和乙环唑暂时缺乏限量标准外,本研究中其他三唑类农药的最大残留限量值均不低于50 μg/kg,因此,本方法能够满足三唑类杀菌剂的监测需求。

将本方法应用于空白浙贝母、白芍、郁金、玄参、麦冬、杭白菊和延胡索等样品基质中三唑类农药残留的分析,添加水平为100 μg/kg,结果见表3。除杭白菊中烯效唑外,7种基质中其余所有农药的回收率在76.4%~123%范围内,RSD均小于12.2%。由此可见,建立的方法具有良好的准确性和精密度,并且对“浙八味”基质具有良好的通用性。

**表3:** 22种农药在浙贝母、白芍、郁金、玄参、麦冬、杭白菊、延胡索基质中的回收率和精密度(*n*=3)

Compound	Recoveries (RSDs)/%							
	Bulbus Fritillariae Thunbergii	Radix paeoniae alba	Curcumae radix	Scrophulariae radix	Ophiopogonis radix	Dendranthema morifolium	Rhizoma corydalis	
Azaconazole	97.8 (1.6)	105 (1.7)	104 (2.7)	102 (0.9)	104 (1.5)	103 (2.3)	97.0 (5.8)	
Bitertanol	101 (2.2)	97.0 (2.5)	103 (1.5)	106 (0.9)	108 (1.6)	111 (5.1)	108 (5.5)	
Bromuconazole	103 (2.9)	106 (2.9)	95.3 (3.9)	105 (2.1)	102 (3.3)	97.1 (4.7)	99.4 (4.6)	
Cyproconazole	102 (1.4)	108 (3.8)	111 (3.0)	109 (1.5)	104 (2.2)	86.6 (2.6)	102 (6.1)	
Difenoconazole	99.8 (0.7)	93.6 (1.3)	86.1 (1.9)	107 (0.7)	100 (2.1)	103 (3.4)	105 (2.0)	
Epoxiconazole	98.4 (2.2)	107 (3.9)	94.6 (1.2)	91.6 (1.8)	109 (4.2)	102 (4.0)	104 (3.0)	
Etaconazole	98.3 (1.3)	108 (1.6)	102 (1.3)	102 (2.7)	105 (1.9)	114 (7.7)	102 (3.7)	
Fenbuconazole	100 (1.6)	98.1 (2.9)	105 (2.1)	107 (1.9)	108 (2.8)	111 (6.7)	108 (3.0)	
Flusilazole	99.9 (1.3)	110 (2.5)	108 (2.4)	106 (1.6)	107 (1.9)	92.5 (2.8)	108 (2.4)	
Flutriafol	95.4 (2.9)	91.5 (5.9)	109 (2.2)	101 (2.8)	110 (3.1)	98.5 (1.4)	100 (2.2)	
Hexaconazole	98.8 (1.9)	104 (1.4)	105 (2.0)	107 (1.3)	104 (2.0)	116 (12)	105 (3.5)	
Ipconazole	97.1 (1.8)	107 (1.7)	90.2 (2.4)	106 (2.0)	101 (1.8)	110 (4.0)	104 (1.8)	
Metconazole	96.0 (0.9)	106 (1.4)	91.2 (1.3)	105 (2.2)	97.1 (1.6)	107 (6.7)	107 (1.6)	
Myclobutanil	102 (1.0)	108 (1.8)	108 (2.3)	106 (0.9)	109 (1.7)	76.4 (5.4)	110 (3.0)	
Penconazole	100 (1.8)	107 (1.2)	106 (2.1)	105 (2.3)	103 (1.1)	115 (2.5)	107 (3.0)	
Propiconazole	102 (1.8)	105 (0.9)	109 (1.7)	104 (1.8)	104 (1.0)	108 (10)	104 (3.2)	
Simeconazole	100 (1.0)	111 (4.8)	117 (1.3)	106 (1.4)	88.5 (10)	118 (3.0)	105 (1.0)	
Tebuconazole	98.1 (0.9)	110 (3.7)	98.4 (1.5)	103 (0.8)	101 (1.0)	104 (1.4)	104 (4.3)	
Tetraconazole	100 (1.2)	108 (2.2)	113 (3.1)	107 (1.3)	110 (1.6)	108 (2.6)	103 (2.9)	
Triadimefon	101 (1.3)	109 (1.5)	107 (2.1)	105 (1.5)	105 (1.2)	112 (1.7)	117 (2.5)	
Triadimenol	98.7 (1.8)	108 (1.7)	109 (2.2)	106 (1.3)	110 (9.4)	108 (1.0)	105 (3.5)	
Uniconazole	93.8 (2.1)	105 (3.3)	91.3 (5.6)	96.3 (4.4)	93.3 (4.5)	123 (4.2)	109 (4.0)	

Spiked level: 100 μg/kg.

### 2.3 与文献方法的对比

经文献调研发现,目前并未有文献对“浙八味”中22种三唑类农药残留的同时分析进行报道,多数文献仍是针对单一基质中少量三唑类农药残留展开的研究,见表4。与文献方法相比,首先,本方法萃取试剂用量较少,尤其是与固相萃取法(SPE)相比,具有消耗溶剂少的优点。其次,本方法展现出良好的灵敏度和准确度。此外,本方法可实现不同基质中22种三唑类农药的检测,具有适用基质种类和药物范围广的优势。因此,本方法展现出良好的应用前景。

**表4:** 本方法与其他方法的比较

Detection method	Matrix	Compound	Pretreatment method	Extractant volume/mL	Recoveries/ %	LOQs/ (μg/kg)	LODs/ (μg/kg)	Ref.
LC-MS/MS	Zhebawei	22 triazole pesticides	QuEChERS	5	77.0-115	10-20	1.0-2.5	this work
GC-MS/MS	Radix Paeoniae Alba	triadimefon	QuEChERS	15	97.6-106	1.7-13.3	0.5-4.0	[24]
		triadimenol			77.8-80.1			
GC-MS/MS	Dendranthema Morifolium	triadimefon	QuEChERS	15	105-111	11.1	3.3	[25]
		triadimenol			100-107	17.1	5.2	
LC-MS/MS	Rhizoma Corydalis	triadimefon	SPE	50	85.4-95.0	18.0	1.2	[26]
GC-MS/MS	Bulbus Fritillariae Thunbergii	propiconazole	QuEChERS	15	-	-	5.0	[27]
UPLC-Q-	Rhizoma Corydalis	bromuconazole	QuEChERS	15	58.0-120	10	-	[28]
Orbitrap HRMS		hexaconazole						
		ipconazole						
		penconazole						
		propiconazole						
		triadimefon						
		triadimenol						

-: not mentioned.

### 2.4 实际样品分析

采用本研究建立的方法对购自市场的30份中药材样品进行检测,包括浙贝母、白芍、麦冬、玄参、郁金、白术、延胡索各3批次和杭白菊9批次,检测结果见表5。共有9个样品检出4种三唑类农药残留,涉及浙贝母和杭白菊2种中药材,其中3个浙贝母样品中均检出苯醚甲环唑,含量为41.4~110 μg/kg, 6个杭白菊样品中检出苯醚甲环唑、腈菌唑、三唑醇和丙环唑,含量为16.1~250 μg/kg。其余样品均未检出三唑类农药。

**表5:** 实际样品中的农药残留含量

Sample	Batch	Difenoco- nazole	Myclob- utanil	Triadim- enol	Propico- nazole
Bulbus	1	94.0			
Fritillariae	2	110			
Thunbergii	3	41.4			
Dendranthema	4		66.0	110	
Morifolium	5	92.00			
	6			30.2	
	7	250	26.0		
	8				16.1
	9			100	

## 3 结论

本研究以MWCNTs-COOH和C18作为净化吸附剂,建立了中草药中22种三唑类杀菌剂农药残留的简便、快速和精准分析方法。以白术为代表性样品展开系统方法优化,筛选出MWCNTs-COOH和C18作为净化吸附剂。方法学考察表明,各目标分析物线性关系良好,准确度和精密度高,分析灵敏度能够满足三唑类杀菌剂的监管需求,本方法对白术、浙贝母、白芍、郁金、玄参、麦冬、杭白菊和延胡索8种基质均展现出良好的适用性,因此可用于“浙八味”中22种三唑类杀菌剂残留的定量分析,为研究三唑类农药在这8种中草药中的污染水平和制定最大残留限量标准提供了方法依据。
